# Optimizing high-throughput viral vector characterization with density gradient equilibrium analytical ultracentrifugation

**DOI:** 10.1007/s00249-023-01654-z

**Published:** 2023-05-02

**Authors:** Shawn M. Sternisha, Abraham D. Wilson, Emilie Bouda, Akash Bhattacharya, Ross VerHeul

**Affiliations:** Beckman Coulter Life Sciences, Indianapolis, IN USA

**Keywords:** AUC, Analytical ultracentrifugation, Density gradient, Adenovirus, Gene therapy

## Abstract

Viral vector-based gene therapies and vaccines require accurate characterization of capsid species. The current gold standard for assessing capsid loading of adeno-associated virus (AAV) is sedimentation velocity analytical ultracentrifugation (SV-AUC). However, routine SV-AUC analysis is often size-limited, especially without the use of advanced techniques (e.g., gravitational-sweep) or when acquiring the multiwavelength data needed for assessing the loading fraction of viral vectors, and requires analysis by specialized software packages. Density gradient equilibrium AUC (DGE-AUC) is a highly simplified analytical method that provides high-resolution separation of biologics of different densities (e.g., empty and full viral capsids). The analysis required is significantly simpler than SV-AUC, and larger viral particles such as adenovirus (AdV) are amenable to characterization by DGE-AUC using cesium chloride gradients. This method provides high-resolution data with significantly less sample (estimated 56-fold improvement in sensitivity compared to SV-AUC). Multiwavelength analysis can also be used without compromising data quality. Finally, DGE-AUC is serotype-agnostic and amenable to intuitive interpretation and analysis (not requiring specialized AUC software). Here, we present suggestions for optimizing DGE-AUC methods and demonstrate a high-throughput AdV packaging analysis with the AUC, running as many as 21 samples in 80 min.

**Supplementary Information:**

The online version contains supplementary material available at 10.1007/s00249-023-01654-z.

## Introduction

Density gradient ultracentrifugation (DGUC) has long been a staple technology providing high-resolution purification of numerous materials used in vaccines, gene therapy, and other advanced therapeutics. While traditionally used for purification, this approach has recently been adapted for the characterization of large biologics, such as viral vectors, using an Optima AUC analytical ultracentrifuge from Beckman Coulter Life Sciences. Meselson and Stahl pioneered using density gradients in the analytical ultracentrifuge (AUC) to elucidate the DNA replication mechanism as early as 1957 (Meselson and Stahl 1958; Meselson et al. 1957), but the technique is undergoing a renaissance today in the field of gene therapy (Yang et al. 2008). Sedimentation velocity-AUC (SV-AUC) is already regarded as the gold-standard characterization technique to determine the empty/partial/full ratio of adeno-associated virus (AAV) particles (Wang et al. 2019), and with the addition of density gradient equilibrium-AUC (DGE-AUC) characterization, the Optima AUC can provide an orthogonal technique to this characterization which has a broader size range than standard SV-AUC experiments (Houde and Berkowitz. 2020). Here, we (1) review development and optimization of the DGE-AUC method, (2) demonstrate a high-throughput implementation with adenovirus (AdV), and (3) highlight advantages of the method.

## Results and discussion

### Method development and optimization

We first optimized the cesium chloride (CsCl) density gradient for characterizing AdV packaging. In isopycnic DGUC, a stable density gradient is formed during centrifugation and particles migrate to a position equal to their buoyant density. The profile of a density gradient at equilibrium is a function of centrifugal force and diffusion; faster rotational speed and lower temperature will result in a steeper gradient, whereas a slower speed and higher temperature will result in a shallower gradient. A steep gradient results in a larger dynamic range of solution densities. As a result, particles concentrate into thinner bands resulting in sharper peaks and high sensitivity. By contrast, shallower gradients result in a smaller dynamic range. Thus, they achieve greater separation between particles of different densities but at the cost of reduced sensitivity (e.g., peak height).

**Fig. 1:**
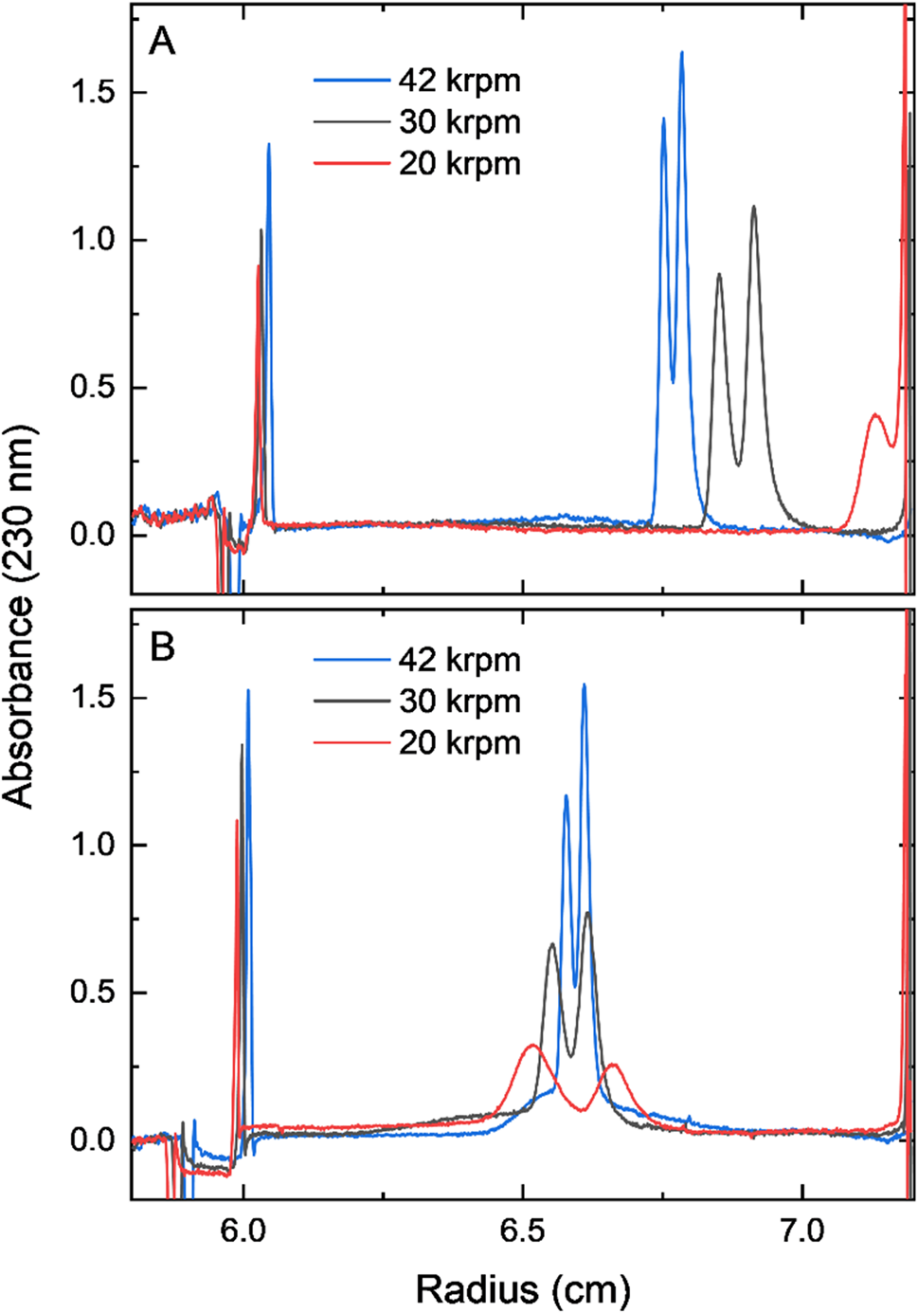
Impact of rotor speed and solution density on AdV analysis via DGE-AUC when CsCl density is **A** 1.30 and **B** 1.325 g/mL; scans shown are at equilibrium (20 °C, 16 h) from a single multi-stage experiment with 2-sector centerpieces

To demonstrate the process of balancing CsCl gradient density and rotor speeds, we optimized these variables for AdV (Fig. 1). We observed that when peaks appear on the same side of the midpoint of the gradient, there is a risk that some sample will sediment (or float) to the bottom (or top) of the solution. Figure 1A shows two major species of AdV in 1.30 g/mL CsCl. Reducing rotor speed from 42 to 20 krpm results in the high-density peak moving off scale and pelleting when the particle density exceeds that of the gradient. However, there is adequate separation between the medium density (or middle) peak and the small, broad low-density shoulder peak, as those two peaks are on opposite sides of the midpoint. In another scenario, the two major species of interest straddle the gradient’s midpoint as in Fig. 1B. In this case, a shallower gradient at reduced speed will move the two major species apart from each other, in opposite directions from the gradient midpoint, thus offering the best possible resolution between those two peaks. This result is only achievable when the starting CsCl solution density is at or near the average of the buoyant density range of the sample species to be distinguished.

We determined the optimal starting CsCl density for AdV by running a DGE-AUC experiment with initial CsCl density varying from 1.30 to 1.35 g/mL in different AUC cells. At 1.325 g/mL, we achieved both excellent peak sensitivity (with the high rotor speed) and near baseline resolution (with the low rotor speed). Notably, the 260/280 nm absorbance ratios for each of the two major peaks are 1.3–1.4, which are in the typical range of genome-loaded particles (Sweeney and Hennessey 2002), while the lower density shoulder peak was too small to reliably fit. Previous work has demonstrated that DGE-AUC can indeed be used to quantify empty and full AdV capsids (Berkowitz and Philo 2007, and the separation of such AdV species by density in CsCl gradients is well documented (Hickey et al. 2022; Takahashi et al. 2006; Xing and Tikoo 2009). The midpoint (hinge point) of a density gradient is the radial position at which the local concentration is equal to the initial cell loading concentration (Harding et al. 2016). Thus, once equilibrium is reached, species (peaks) appearing on the left of the midpoint will have buoyant densities lower than the initial cell loading density and peaks appearing to the right of the midpoint will have buoyant densities higher than the starting solution density. In these AdV experiments, the two peaks appeared on the right of the gradient midpoint when the initial solution density was 1.30 g/mL (Figure S1), to the left of the midpoint when the initial solution density was 1.35 g/mL (Figure S2), and near the midpoint itself when the initial solution density was 1.325 g/mL (Fig. 1B). These data indicate that the two peaks are likely two species with different amounts of DNA since AdV capsids devoid of nucleic acid display a buoyant density of 1.29–1.30 g/mL (Edvardsson et al. 1976). On the basis of prior studies, measured 260/280 ratios, positioning of the peaks at various initial loading concentrations of CsCl, and the fact that these samples were purified using CsCl gradients and, therefore, should not have a consequential number of empty capsids, the two major peaks are expected to be populations of loaded virus particles.

We recommend using Table 1 as a guide to quickly optimize a DGE-AUC experiment for a new sample type. In Phase 1 of screening, the objective is to determine (1) the approximate starting CsCl density, (2) the time required to reach equilibrium, and (3) the sample quantity required to achieve sufficient signal. Next, in Phase 2, evaluating 2 or 3 rotor speeds and a finer range of CsCl densities enables selection of the ideal density where species can be maximally resolved. Finally, Phase 3 consists of a multispeed experiment with the optimized CsCl density to select the ideal balance of sensitivity and resolution. Following these three phases of optimization, the finalized protocol will only have a single CsCl starting density and rotor speed which generates sufficient data for easy quantification of the different biologics in the sample. Of course, earlier screening phases may be skipped or truncated with prior knowledge of a particle’s buoyant density.

**Table 1:** Suggested conditions in a three-phase exemplar workflow to optimize a DGE-AUC method

Phase	Stages	CsCl density (g/mL)	Rotor speed (krpm)	Runtime (h)
1	1	1.20–1.40	42	24
2	2	1.30–1.35	42	16
			25	8
3	6	1.325	42	16
			40	8
			35	8
			30	8
			25	8
			20	8

For this work, we utilized an 8-hole An-50 Ti rotor, however, higher speeds resulting in steeper gradients and shorter equilibration times can be achieved with a 4-hole An-60 Ti rotor and aluminum centerpieces, though the benefits of screening more conditions in a single run can outweigh these time savings. Note that a long radial pathlength (e.g., with a standard 2-sector centerpiece) is ideal for initial screening to increase the gradient range and reduce the likelihood of the particles of interest floating or pelleting out of solution. We maintained a fixed temperature and fill volume (e.g., 400–440 µL) throughout the screening process. Unless working at the extremes of the instrument’s accessible temperature range, a temperature equilibration stage is often not required as the temperature will have equilibrated before sample equilibrium is reached. Finally, we recommend loading a reference sample of equivalent volume (including density-matched CsCl) to ensure the sample and reference density gradients are equivalent.

The CsCl gradient, due to the significant change in refractive index, can be directly monitored with Rayleigh interference while the sample movement and equilibrium position can be analyzed with UV/Vis absorbance. Experiments using only the density gradient forming material can be used for extensive preliminary optimization (e.g., time to reach equilibrium) to reduce the financial burden associated with running costly viral vectors. Through the screening process, collecting scans at 5–10 min intervals with both absorbance and interference typically provides sufficient information to understand the approach to equilibrium and make informed decisions.

The process of optimizing the starting CsCl density can also yield information about low-abundance species, as seen in Fig. 1b. The low-abundance, low-density species appears as a shoulder peak at 6.55 cm when the starting CsCl density is 1.325 g/ml, but is seen as a broad and low, but distinct peak at 6.45 cm in Fig. 1a, where the starting CsCl density is 1.3 g/ml. This suggests that slightly offset CsCl densities can reveal the presence of low-abundance species which would not be easily visible in the optimized final experiment. These low-abundance species can be studied by increasing sample concentration, even past the point where the main species peaks will likely saturate the detector.

### Optimizing throughput

**Fig. 2:**
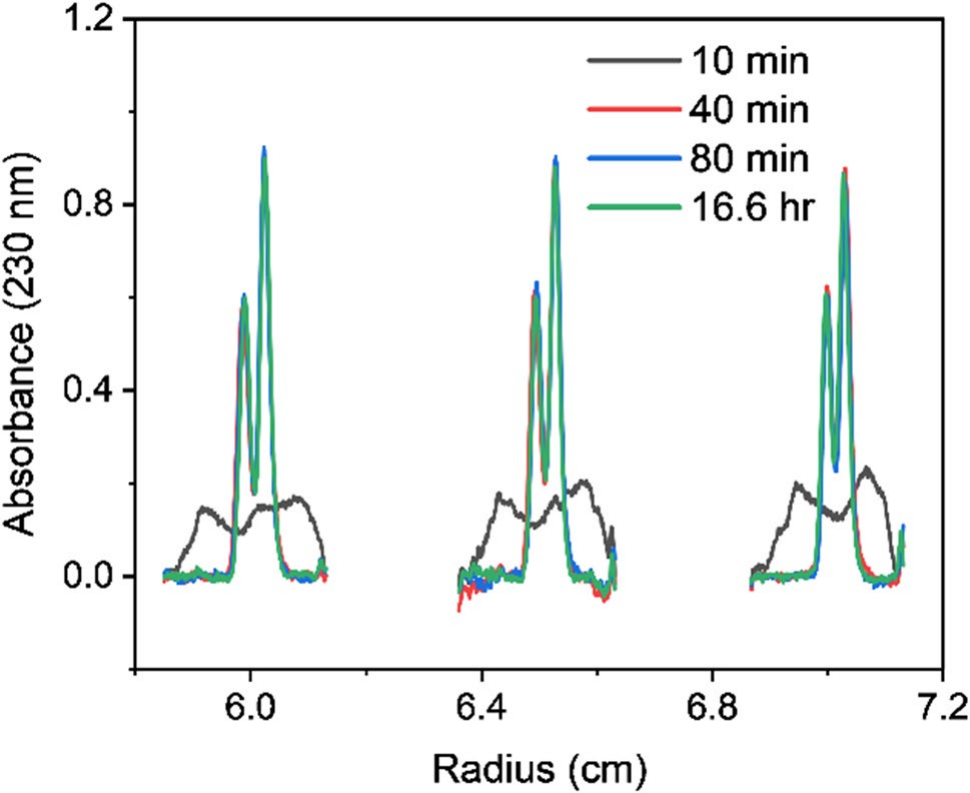
High-throughput DGE-AUC analysis of AdV with equilibrium achieved in 80 min (42 krpm, 20 °C) using a 6-channel centerpiece

One key advantage of density gradient equilibrium experiments is the opportunity to use equilibrium 6-channel centerpieces, allowing for the characterization of 21 unique samples simultaneously (or 42 if conducting intensity-mode experiments) (Savelyev et al. 2020). Furthermore, the shorter radial pathlength of the 6-channel centerpiece provides the additional advantage of requiring significantly less time to reach equilibrium. For these experiments, 42 krpm was chosen as we expected that speed to afford rapid equilibration while still maintaining adequate peak separation. With AdV as an example, sample equilibrium was reached in 80 min (Fig. 2), where the average difference between individual peak areas at 16.6 h was 2.1%. Similarly, equilibrium was nearly achieved in 40 min, with an average difference in peak areas of 7.8%. On this premise, it may be possible to conduct 5 runs and analyze more than 100 samples in a single 8-h shift (assuming room temperature is selected). Results of peak area comparisons across time points are summarized in Table 2.

**Table 2:** Quantifying time to equilibrium in DGE using 6-channel centerpieces (20 °C, 42 krpm)

Time point	% Difference in peak area from 16.6 h equilibrium time point (230 nm)
10 min	18.0
40 min	7.8
80 min	2.1
16.6 h	0

When using the 6-channel centerpieces, the profile of the density gradient that forms in the inner channels and outer channels will differ as a result of the difference in rotational acceleration associated with varying radial positions. Notwithstanding these slight gradient differences, Fig. 2 demonstrates that all three sample channels in a 6-channel centerpiece show consistent, excellent separation for the two major AdV species when using the same 1.325 g/mL density for all channels.

### Advantages of DGE-AUC and future potential

In addition to the higher potential throughput, there are many other beneficial implications of a density-based analytical method. First, the data are easily relatable as the interpretation is analogous to industry-standard preparative CsCl DGUC. Since physical separation is achieved with DGE-AUC, computational deconvolution of species is typically not required, and analysis of the peaks is direct and intuitive. Analysis requires no AUC-specific software or supercomputers, and complete analysis of a sample in less than 1 min is possible using OriginPro 2022 software. Notably, improved quantitative accuracy can be realized when using the 2-sector centerpieces by applying a weighted correction factor to account for the sector-shaped geometry (Saelyev et al. 2023). The volume of each radial segment can be calculated and multiplied by the corresponding absorbance to correct for the sector’s progressively increasing volume. Doing so can also allow for determination of the number of particles in a given volume (i.e., peak). Importantly, 6-channel centerpieces are not sector shaped and do not need such geometric compensation. Like DGUC, DGE-AUC is a robust, density-resolved separation method known to work universally for any serotype and even very large particles. Simultaneously, DGE-AUC can provide high-resolution data with approximately 56-fold higher sensitivity, and high concentration samples can be loaded to detect low-abundance species at different radial positions. DGE-AUC is not time-resolved; thus, the time required to observe many absorbance wavelengths will not impact resolution. This opens the possibility to selectively track multiple distinct chromophores with different absorbance wavelengths. Further, after the sample has reached thermodynamic equilibrium, scan replicates can be collected and averaged for the highest possible sensitivity and signal–noise ratio. Finally, studies have shown how radial position can be correlated with density to obtain another layer of information for particles of interest (Ifft et al. 1961). For example, a particle’s partial specific volume (v̄), which is an important parameter used in many biophysical studies, is equal to the inverse of its position in the CsCl gradient. Notably, the v̄ measured in CsCl is different (usually smaller) than that measured in simple aqueous solutions like PBS as a result of preferential solvation.

To conclude, DGE-AUC is a promising high-throughput technique complementary to SV-AUC. DGE-AUC may be capable of screening in-process samples with appropriate assay optimization (i.e., to minimize the impact of in-process impurities) when time and sample are in limited supply. Critically, while SV-AUC and DGE-AUC may both be used to answer the same analytical questions, they are based on fundamentally different principles (SV characterizes by sedimentation rate while DGE characterizes by buoyant density). Thus, AUC as a technology provides two orthogonal techniques useful in characterizing loading of viral vectors and other particles.

## Materials and methods

### AdV production

Purified AdV particles were purchased from Sirion Biotech (Graefelfing, Germany). Briefly, adherent HEK-293 were seeded at 1.5 × 10^8^ (4 × 550 mL cultures) and transduced at a multiplicity of infection (MOI) of 5 using pAd5-CMV-eGFP-bGHpA. Viral production was carried out for 48 h and cells were harvested via centrifugation at 600 × *g* for 20 min at 4 °C, and then resuspended in 32 mL fresh growth medium (8 mL per culture pellet). The resulting suspension was spiked with sodium deoxycholate (0.25% w/v), MgCl_2_ (20 mM), and DNAse I (50 µg/mL) and incubated for 20 min at 37 °C (shown are final working concentrations). The lysate was clarified via centrifugation at 4500 × *g* for 10 min at 4 °C. 13.2 mL Open-Top Thinwall Ultra-Clear Tubes (Beckman Coulter; PN 344059) were layered with 3 mL of 1.41 g/mL CsCl and then 5 mL of 1.27 g/mL CsCl. The tubes were filled to approximately 2–3 mm from the top with clarified lysate (~ 4 mL) and loaded into a SW 41 Ti swinging-bucket rotor (Beckman Coulter). The rotor was then loaded into an Optima L-70 ultracentrifuge (Beckman Coulter) and spun at 119,000 × *g* for 90 min at 4 °C. The tubes were carefully removed from the rotor and a 2 mL syringe with a 20 gauge (0.90 × 40 mm) needle was used to pierce the side of the tubes and extract the visible virus layer (1–2 mL extracted from each tube). To remove CsCl and exchange samples into an appropriate buffer, disposable PD10 desalting columns (Cytiva) were equilibrated with 1 × HEPES buffer and manufacturer’s instructions were followed. Briefly, purified virus sample was loaded onto the column and allowed to run out completely. Viral particles were eluted with 1 × HEPES buffer (pH 7.4) and 7 fractions were collected; selected fractions were then pooled based on 260 nm absorbance. Sucrose was added to a final concentration of 4% w/v and the virus sample was aliquoted and frozen at − 80 °C until use. We do not anticipate meaningful contribution of sucrose to solution density as greater than 100-fold dilutions were used for DGE-AUC experiments, resulting in final sucrose concentrations of 0.04% w/v or lower.

### DGE-AUC

For DGE-AUC experiments, we first prepared a high-density (1.720 g/mL) stock solution of optical grade CsCl in deionized water and verified density by refractive index (1.4012). We prepared samples for DGE-AUC analysis by mixing purified AdV, the stock solution of CsCl, 10 × PBS (pH 7.4), and deionized water at calculated ratios to generate a sample with defined characteristics including 230 nm absorption (using 10 mm pathlength) equal to 0.16; solution density equal to 1.30 or 1.35 g/mL, and buffer concentration equal to 1 × PBS. For 2-sector experiments, we loaded 420 µL of sample into the sample sector and 420 µL of a CsCl solution at equivalent density into the reference sector of a cell assembly containing a 12 mm epon charcoal-filled 2-sector centerpiece and sapphire windows (Beckman Coulter; PN 392773). For 6-channel experiments, we loaded 120 µL of sample into each sample sector and 120 µL of a CsCl solution at equivalent density into each reference sector of cell assembly containing a 12 mm epon charcoal-filled 6-channel centerpiece and sapphire windows (Beckman Coulter; PN A37297). Sealed and torqued cells were loaded and aligned in an 8-hole An-50 Ti rotor which was then placed into an Optima AUC (A/I) according to the rotor and instrument instructions for use. Using the 2-sector centerpieces, we ran a multispeed experiment from 42 to 5 krpm (42, 40, 35, 30, 25, 20, 15, 10, and 5 krpm) at 20 °C where each stage consisted of interference and 230, 260, and 280 nm absorbance detection with 100 scans each at 600 s intervals. Using the 6-channel centerpieces, we ran a multispeed experiment at 42, 35, and 25 krpm and 20 °C with interference and 230, 260, and 280 nm absorbance detection (100 scans at 600 s intervals per stage). We downloaded the data from the Optima AUC and imported it directly into OriginPro 2022 (OriginLabs, MA, USA), where built-in features including baseline correction and peak integration were used according to the manufacturer’s instructions.

To estimate technique sensitivity, peak maxima were calculated and summed for DGE-AUC and compared to total signal (i.e., plateau) in SV-AUC raw data. 2-sector centerpiece data were used for comparison. The starting absorbance (12 mm pathlength) for the SV-AUC experiment was 0.55 after subtracting out baseline signal. For the DGE-AUC experiment with the same sample, the starting absorbance was 0.16, which yielded a summed peak height/intensity of 3.05 after analysis. The DGE-AUC sample was diluted tenfold over the SV-AUC sample so multiplying the dilution factor 10 by the total signal height of 3.05 equals 30.5. Dividing this estimate of sensitivity by the total signal of 0.55 for SV-AUC results in 55.5. Thus, the estimated increase in sensitivity of DGE experiments versus SV experiments is approximately 56-fold. Importantly, this estimate should not be confused with comprehensive determination of limit of detection (LOD) and limit of quantification (LOQ). Processed SV-AUC data were not used as the AdV sample sedimented too quickly to acquire reliable data regardless of speed.

## Supplementary Information

Below is the link to the electronic supplementary material.

Supplementary file1 (DOCX 283 KB)

## Data Availability

OriginPro 2023 is available for purchase or free trial at https://www.originlab.com. All the AUC data are available in the manuscript.

## Declarations

### Legal notices

Origin 2022 and OriginPro 2022 (both produced by the OriginLab Corporation) have been used for the analyses of the raw data generated by the Beckman Coulter Inc. (“Beckman”) Analytical Ultracentrifuge. This third-party analysis software has not been validated by Beckman for use with the Beckman Analytical Ultracentrifuge. Beckman does not endorse any third-party analyses software. Beckman warranty and/or performance guarantee that may be applicable or are provided by Beckman for Beckman Analytical Ultracentrifuge do not apply to any third-party software. Copyright, License and Terms of Use Disclaimers are documented for the below software on their respective pages. OriginLab: https://www.originlab.com/index.aspx?go=Company/TermsOfUse.

### Regulatory/Trademark statements

All products and services identified, unless noted as for in vitro diagnostic (IVD) use, are for research use and not intended or validated for use in the diagnosis of disease or other conditions. © 2022 Beckman Coulter, Inc. All rights reserved. Beckman Coulter, the stylized logo, and the Beckman Coulter product and service marks used herein are trademarks or registered trademarks of Beckman Coulter, Inc. in the United States and other countries. All other trademarks are the property of their respective owners.
