# Supplementary material for: Optimizing high-throughput viral vector characterization with density gradient equilibrium analytical ultracentrifugation
Source: Eur Biophys J. 2023 May 2;52(4-5):387–92. doi: 10.1007/s00249-023-01654-z (PMC10444642; doi:10.1007/s00249-023-01654-z)

Supplementary Information

Optimizing high-throughput viral vector characterization with density gradient equilibrium analytical ultracentrifugation

*Shawn M. Sternisha*, Abraham Dow Wilson, Emilie Bouda, Akash Bhattacharya, Ross VerHeul*

Beckman Coulter Life Sciences, USA

*Corresponding author: [shawnsternisha@gmail.com](mailto:shawnsternisha@gmail.com)

**Figure S1.** AdV approach to equilibrium via DGE-AUC with initial solution density of 1.30 g/mL. A standard Epon-charcoal 2-sector centerpiece was used. No changes are observed after ~100 minutes. The legend shows scan number (10 min scan frequency).


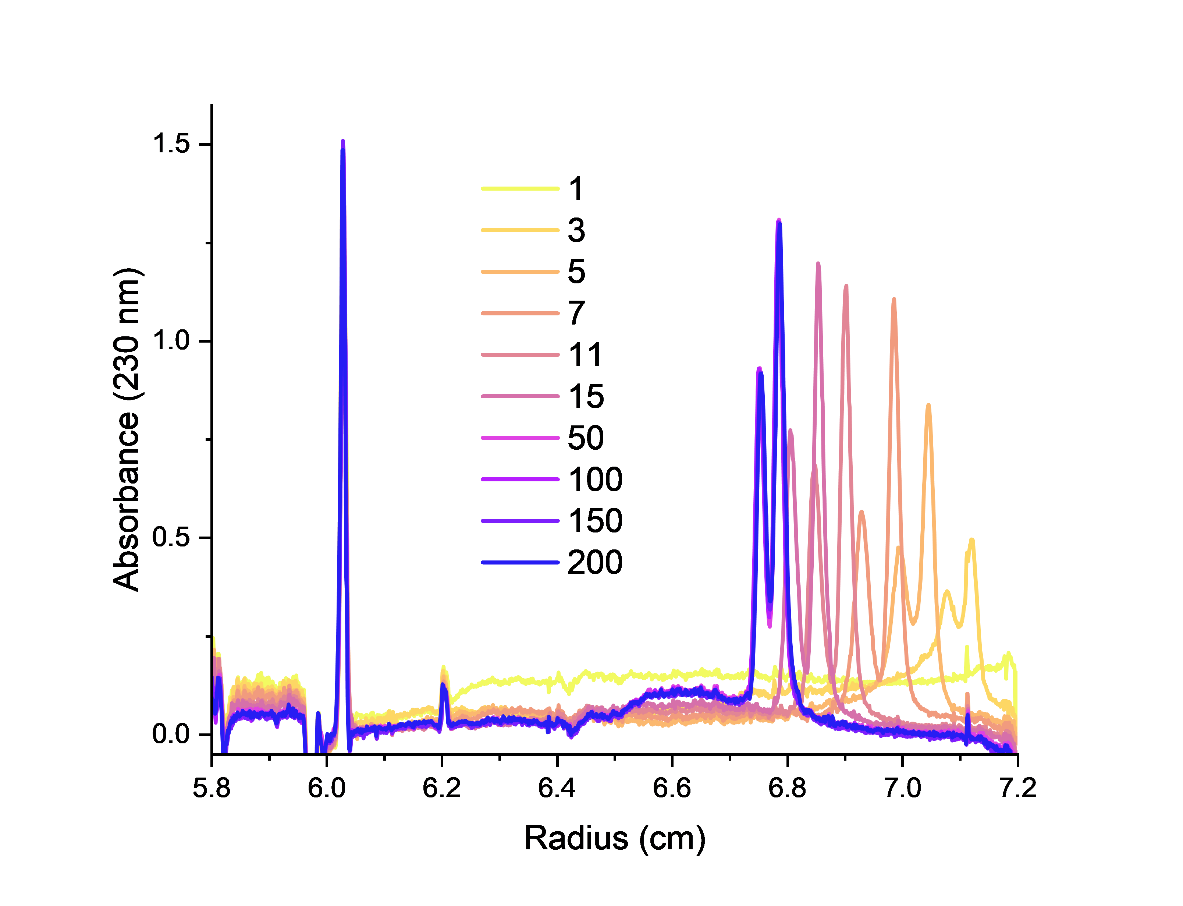


**Figure S2.** AdV approach to equilibrium via DGE-AUC with initial solution density of 1.35 g/mL. A standard Epon-charcoal 2-sector centerpiece was used. No changes are observed after ~100 minutes. The legend shows scan number (10 min scan frequency).


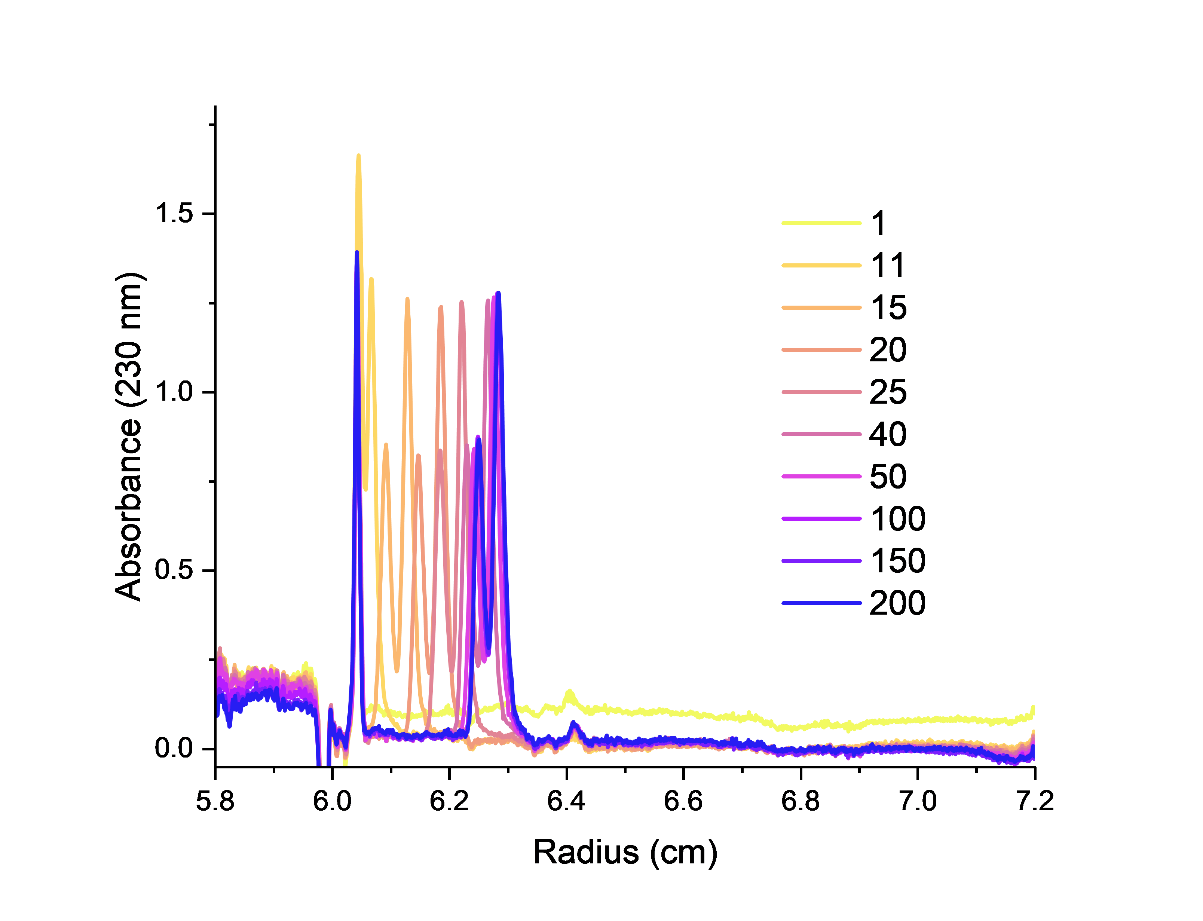

Supplement: Supplementary file 1 — Supplementary file1 (DOCX 283 KB) [file 249_2023_1654_MOESM1_ESM.docx]
